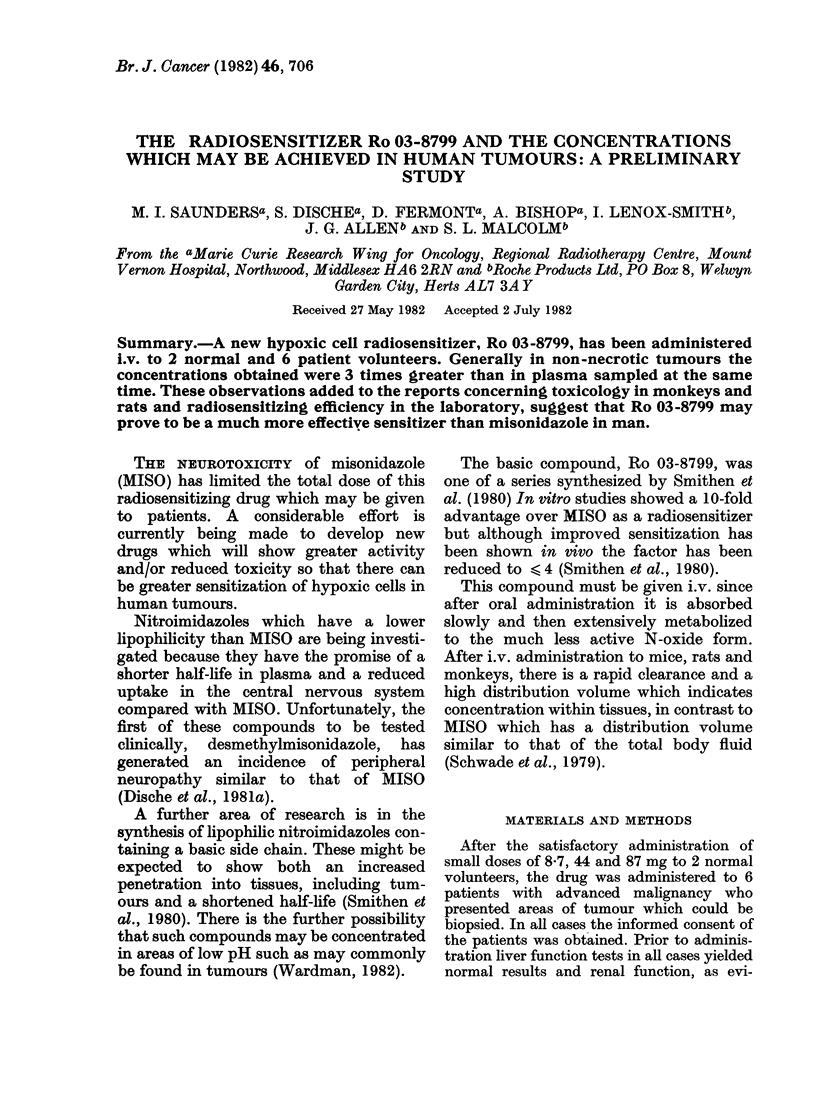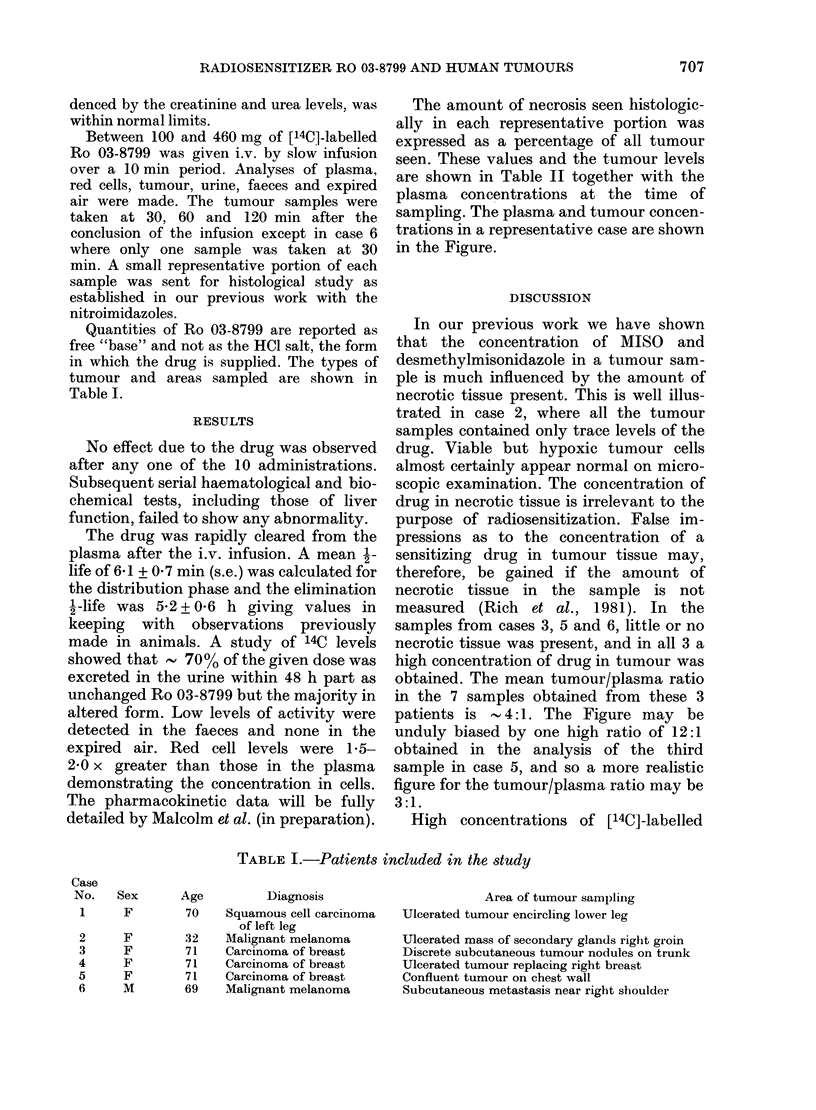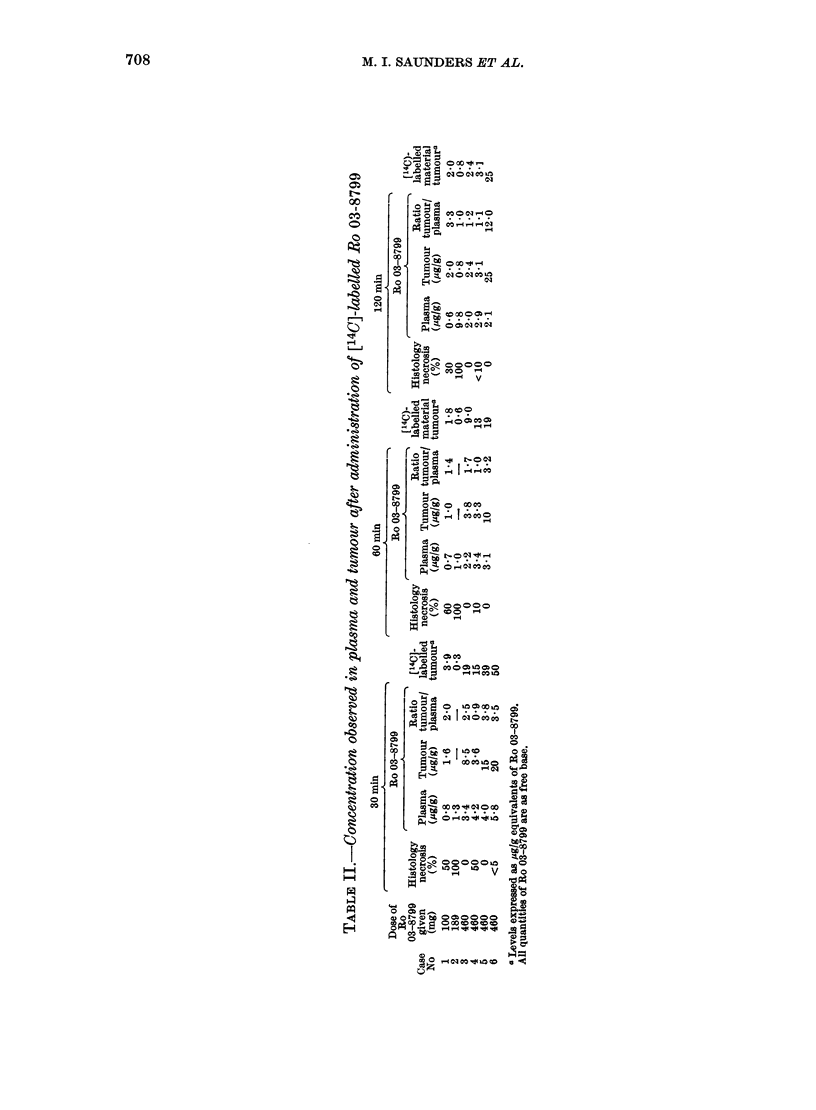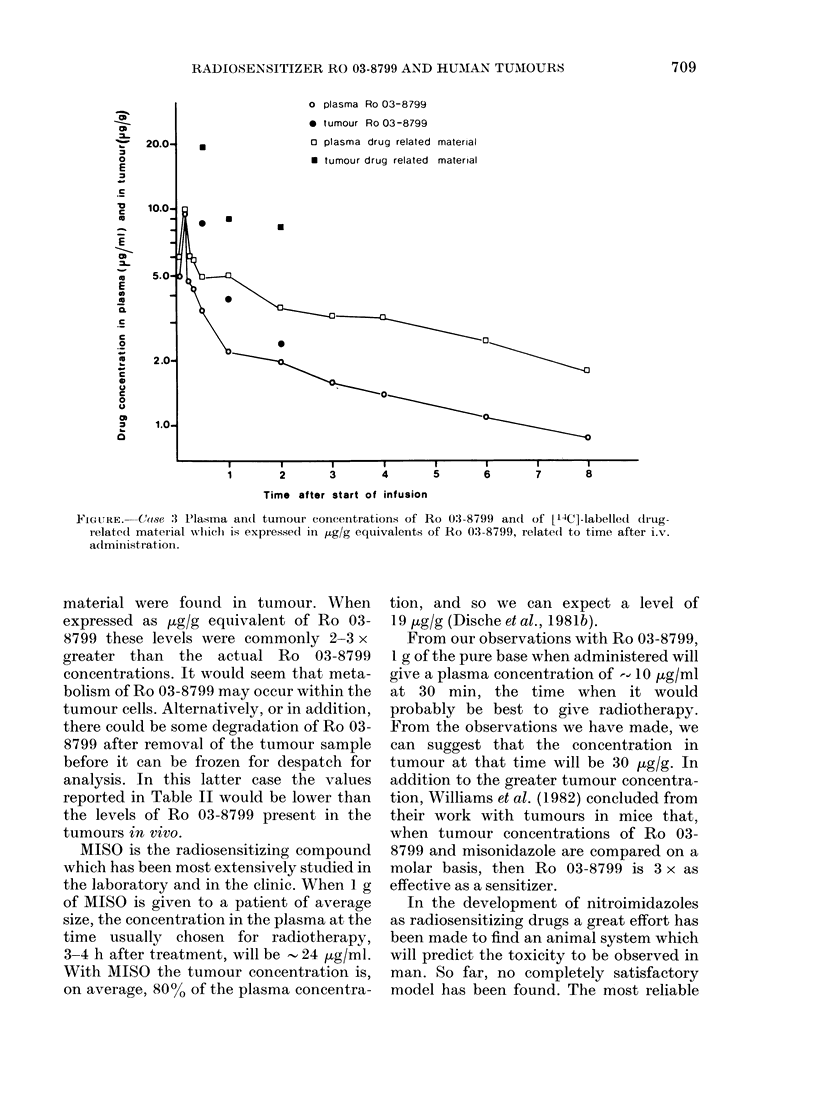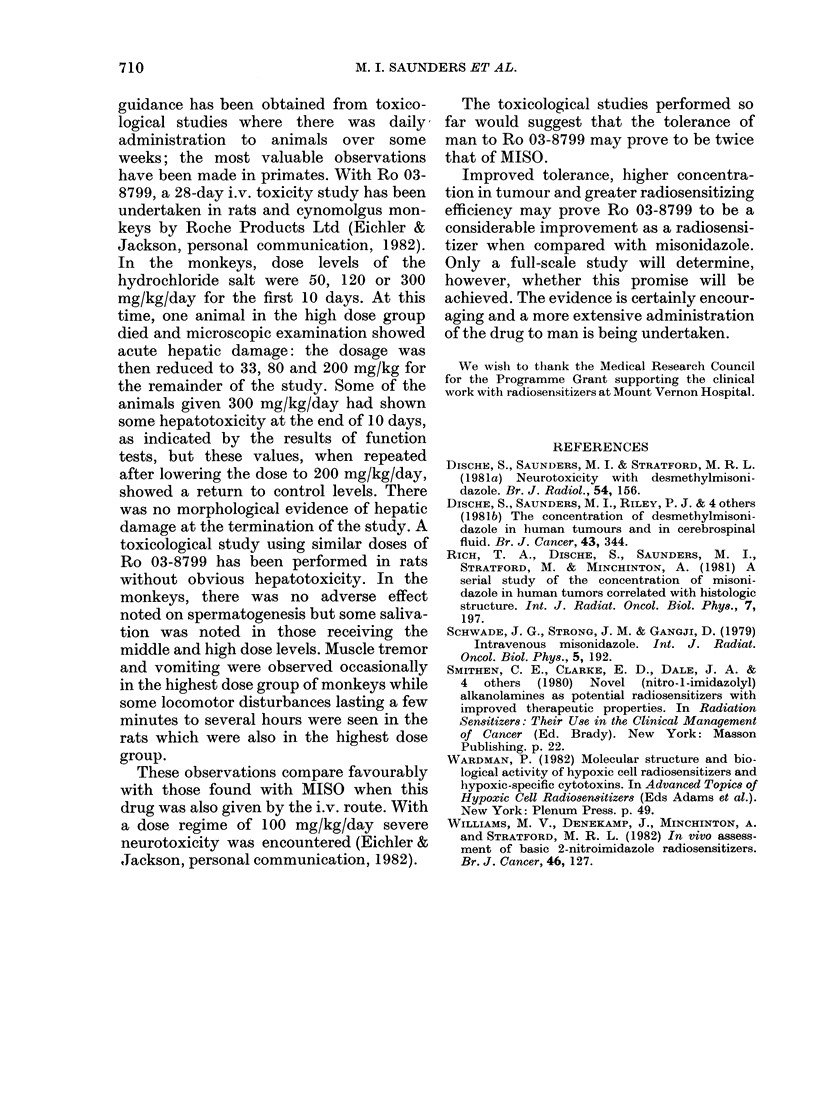# The radiosensitizer Ro 03-8799 and the concentrations which may be achieved in human tumours: a preliminary study.

**DOI:** 10.1038/bjc.1982.262

**Published:** 1982-11

**Authors:** M. I. Saunders, S. Dische, D. Fermont, A. Bishop, I. Lenox-Smith, J. G. Allen, S. L. Malcolm

## Abstract

A new hypoxic cell radiosensitizer, Ro 03-8799, has been administered i.v. to 2 normal and 6 patient volunteers. Generally in non-necrotic tumours the concentrations obtained were 3 times greater than in plasma sampled at the same time. These observations added to the reports concerning toxicology in monkeys and rats and radiosensitizing efficiency in the laboratory, suggest that Ro 03-8799 may prove to be much more effective sensitizer than misonidazole in man.


*Br. J. Cancer* (1982) **46**, 706

### THE RADIOSENSITIZER Ro 03-8799 AND THE CONCENTRATIONS WHICH MAY BE ACHIEVED IN HUMAN TUMOURS: A PRELIMINARY STUDY

M. I. SAUNDERS[a], S. DISCHE[a], D. FERMONT[a], A. BISHOP[a], I. LENOX-SMITH[b], J. G. ALLEN[b] AND S. L. MALCOLM[b]

*From the [a]Marie Curie Research Wing for Oncology, Regional Radiotherapy Centre, Mount Vernon Hospital, Northwood, Middlesex HA6 2RN and [b]Roche Products Ltd, PO Box 8, Welwyn Garden City, Herts AL7 3AY*

Received 27 May 1982 Accepted 2 July 1982

**Summary.—A new hypoxic cell radiosensitizer, Ro 03-8799, has been administered i.v. to 2 normal and 6 patient volunteers. Generally in non-necrotic tumours the concentrations obtained were 3 times greater than in plasma sampled at the same time. These observations added to the reports concerning toxicology in monkeys and rats and radiosensitizing efficiency in the laboratory, suggest that Ro 03-8799 may prove to be a much more effective sensitizer than misonidazole in man.**

THE NEUROTOXICITY of misonidazole (MISO) has limited the total dose of this radiosensitizing drug which may be given to patients. A considerable effort is currently being made to develop new drugs which will show greater activity and/or reduced toxicity so that there can be greater sensitization of hypoxic cells in human tumours.

Nitroimidazoles which have a lower lipophilicity than MISO are being investigated because they have the promise of a shorter half-life in plasma and a reduced uptake in the central nervous system compared with MISO. Unfortunately, the first of these compounds to be tested clinically, desmethylmisonidazole, has generated an incidence of peripheral neuropathy similar to that of MISO (Dische *et al.*, 1981*a*).

A further area of research is in the synthesis of lipophilic nitroimidazoles containing a basic side chain. These might be expected to show both an increased penetration into tissues, including tumours and a shortened half-life (Smithen *et al.*, 1980). There is the further possibility that such compounds may be concentrated in areas of low pH such as may commonly be found in tumours (Wardman, 1982).

The basic compound, Ro 03-8799, was one of a series synthesized by Smithen *et al.* (1980) *In vitro* studies showed a 10-fold advantage over MISO as a radiosensitizer but although improved sensitization has been shown *in vivo* the factor has been reduced to $\leqslant 4$ (Smithen *et al.*, 1980).

This compound must be given i.v. since after oral administration it is absorbed slowly and then extensively metabolized to the much less active N-oxide form. After i.v. administration to mice, rats and monkeys, there is a rapid clearance and a high distribution volume which indicates concentration within tissues, in contrast to MISO which has a distribution volume similar to that of the total body fluid (Schwade *et al.*, 1979).

### MATERIALS AND METHODS

After the satisfactory administration of small doses of 8·7, 44 and 87 mg to 2 normal volunteers, the drug was administered to 6 patients with advanced malignancy who presented areas of tumour which could be biopsied. In all cases the informed consent of the patients was obtained. Prior to administration liver function tests in all cases yielded normal results and renal function, as evidenced by the creatinine and urea levels, was within normal limits.

Between 100 and 460 mg of [$^{14}$C]-labelled Ro 03-8799 was given i.v. by slow infusion over a 10 min period. Analyses of plasma, red cells, tumour, urine, faeces and expired air were made. The tumour samples were taken at 30, 60 and 120 min after the conclusion of the infusion except in case 6 where only one sample was taken at 30 min. A small representative portion of each sample was sent for histological study as established in our previous work with the nitroimidazoles.

Quantities of Ro 03-8799 are reported as free "base" and not as the HCl salt, the form in which the drug is supplied. The types of tumour and areas sampled are shown in Table I.

### RESULTS

No effect due to the drug was observed after any one of the 10 administrations. Subsequent serial haematological and biochemical tests, including those of liver function, failed to show any abnormality.

The drug was rapidly cleared from the plasma after the i.v. infusion. A mean $\frac{1}{2}$-life of $6 \cdot 1 \pm 0 \cdot 7$ min (s.e.) was calculated for the distribution phase and the elimination $\frac{1}{2}$-life was $5 \cdot 2 \pm 0 \cdot 6$ h giving values in keeping with observations previously made in animals. A study of $^{14}$C levels showed that $\sim$ 70% of the given dose was excreted in the urine within 48 h part as unchanged Ro 03-8799 but the majority in altered form. Low levels of activity were detected in the faeces and none in the expired air. Red cell levels were 1·5–2·0 × greater than those in the plasma demonstrating the concentration in cells. The pharmacokinetic data will be fully detailed by Malcolm *et al.* (in preparation).

The amount of necrosis seen histologically in each representative portion was expressed as a percentage of all tumour seen. These values and the tumour levels are shown in Table II together with the plasma concentrations at the time of sampling. The plasma and tumour concentrations in a representative case are shown in the Figure.

### DISCUSSION

In our previous work we have shown that the concentration of MISO and desmethylmisonidazole in a tumour sample is much influenced by the amount of necrotic tissue present. This is well illustrated in case 2, where all the tumour samples contained only trace levels of the drug. Viable but hypoxic tumour cells almost certainly appear normal on microscopic examination. The concentration of drug in necrotic tissue is irrelevant to the purpose of radiosensitization. False impressions as to the concentration of a sensitizing drug in tumour tissue may, therefore, be gained if the amount of necrotic tissue in the sample is not measured (Rich *et al.*, 1981). In the samples from cases 3, 5 and 6, little or no necrotic tissue was present, and in all 3 a high concentration of drug in tumour was obtained. The mean tumour/plasma ratio in the 7 samples obtained from these 3 patients is $\sim$4:1. The Figure may be unduly biased by one high ratio of 12:1 obtained in the analysis of the third sample in case 5, and so a more realistic figure for the tumour/plasma ratio may be 3:1.

High concentrations of [$^{14}$C]-labelled

TABLE I.—*Patients included in the study*

| Case No. | Sex | Age | Diagnosis | Area of tumour sampling |
|---|---|---|---|---|
| 1 | F | 70 | Squamous cell carcinoma of left leg | Ulcerated tumour encircling lower leg |
| 2 | F | 32 | Malignant melanoma | Ulcerated mass of secondary glands right groin |
| 3 | F | 71 | Carcinoma of breast | Discrete subcutaneous tumour nodules on trunk |
| 4 | F | 71 | Carcinoma of breast | Ulcerated tumour replacing right breast |
| 5 | F | 71 | Carcinoma of breast | Confluent tumour on chest wall |
| 6 | M | 69 | Malignant melanoma | Subcutaneous metastasis near right shoulder |

TABLE II.—*Concentration observed in plasma and tumour after administration of* [$^{14}$*C*]-*labelled Ro* 03-8799

| | | 30 min | | | | | 60 min | | | | | 120 min | | | | |
|---|---|---|---|---|---|---|---|---|---|---|---|---|---|---|---|---|
| | | | Ro 03–8799 | | | | | Ro 03–8799 | | | | | Ro 03–8799 | | | |
| Case No | Dose of Ro 03–8799 given (mg) | Histology necrosis (%) | Plasma ($\mu$g/g) | Tumour ($\mu$g/g) | Ratio tumour/ plasma | [$^{14}$C]-labelled tumour[a] | Histology necrosis (%) | Plasma ($\mu$g/g) | Tumour ($\mu$g/g) | Ratio tumour/ plasma | [$^{14}$C)-labelled material tumour[a] | Histology necrosis (%) | Plasma ($\mu$g/g) | Tumour ($\mu$g/g) | Ratio tumour/ plasma | [$^{14}$C)-labelled material tumour[a] |
| 1 | 100 | 50 | 0·8 | 1·6 | 2·0 | 3·9 | 60 | 0·7 | 1·0 | 1·4 | 1·8 | 30 | 0·6 | 2·0 | 3·3 | 2·0 |
| 2 | 189 | 100 | 1·3 | — | — | 0·3 | 100 | 1·0 | — | — | 0·6 | 100 | 9·8 | 0·8 | 1·0 | 0·8 |
| 3 | 460 | 0 | 3·4 | 8·5 | 2·5 | 19 | 0 | 2·2 | 3·8 | 1·7 | 9·0 | 0 | 2·0 | 2·4 | 1·2 | 2·4 |
| 4 | 460 | 50 | 4·2 | 3·6 | 0·9 | 15 | 10 | 3·4 | 3·3 | 1·0 | 13 | < 10 | 2·9 | 3·1 | 1·1 | 3·1 |
| 5 | 460 | 0 | 4·0 | 15 | 3·8 | 39 | 0 | 3·1 | 10 | 3·2 | 19 | 0 | 2·1 | 25 | 12·0 | 25 |
| 6 | 460 | <5 | 5·8 | 20 | 3·5 | 50 | | | | | | | | | | |

[a] Levels expressed as $\mu$g/g equivalents of Ro 03–8799.

All quantities of Ro 03–8799 are as free base.

FIGURE.—*Case* 3 Plasma and tumour concentrations of Ro 03-8799 and of [$^{14}$C]-labelled drug-related material which is expressed in μg/g equivalents of Ro 03-8799, related to time after i.v. administration.

material were found in tumour. When expressed as μg/g equivalent of Ro 03-8799 these levels were commonly 2–3 × greater than the actual Ro 03-8799 concentrations. It would seem that metabolism of Ro 03-8799 may occur within the tumour cells. Alternatively, or in addition, there could be some degradation of Ro 03-8799 after removal of the tumour sample before it can be frozen for despatch for analysis. In this latter case the values reported in Table II would be lower than the levels of Ro 03-8799 present in the tumours *in vivo*.

MISO is the radiosensitizing compound which has been most extensively studied in the laboratory and in the clinic. When 1 g of MISO is given to a patient of average size, the concentration in the plasma at the time usually chosen for radiotherapy, 3–4 h after treatment, will be ~24 μg/ml. With MISO the tumour concentration is, on average, 80% of the plasma concentration, and so we can expect a level of 19 μg/g (Dische *et al.*, 1981*b*).

From our observations with Ro 03-8799, 1 g of the pure base when administered will give a plasma concentration of ~10 μg/ml at 30 min, the time when it would probably be best to give radiotherapy. From the observations we have made, we can suggest that the concentration in tumour at that time will be 30 μg/g. In addition to the greater tumour concentration, Williams *et al.* (1982) concluded from their work with tumours in mice that, when tumour concentrations of Ro 03-8799 and misonidazole are compared on a molar basis, then Ro 03-8799 is 3 × as effective as a sensitizer.

In the development of nitroimidazoles as radiosensitizing drugs a great effort has been made to find an animal system which will predict the toxicity to be observed in man. So far, no completely satisfactory model has been found. The most reliable guidance has been obtained from toxicological studies where there was daily administration to animals over some weeks; the most valuable observations have been made in primates. With Ro 03-8799, a 28-day i.v. toxicity study has been undertaken in rats and cynomolgus monkeys by Roche Products Ltd (Eichler & Jackson, personal communication, 1982). In the monkeys, dose levels of the hydrochloride salt were 50, 120 or 300 mg/kg/day for the first 10 days. At this time, one animal in the high dose group died and microscopic examination showed acute hepatic damage: the dosage was then reduced to 33, 80 and 200 mg/kg for the remainder of the study. Some of the animals given 300 mg/kg/day had shown some hepatotoxicity at the end of 10 days, as indicated by the results of function tests, but these values, when repeated after lowering the dose to 200 mg/kg/day, showed a return to control levels. There was no morphological evidence of hepatic damage at the termination of the study. A toxicological study using similar doses of Ro 03-8799 has been performed in rats without obvious hepatotoxicity. In the monkeys, there was no adverse effect noted on spermatogenesis but some salivation was noted in those receiving the middle and high dose levels. Muscle tremor and vomiting were observed occasionally in the highest dose group of monkeys while some locomotor disturbances lasting a few minutes to several hours were seen in the rats which were also in the highest dose group.

These observations compare favourably with those found with MISO when this drug was also given by the i.v. route. With a dose regime of 100 mg/kg/day severe neurotoxicity was encountered (Eichler & Jackson, personal communication, 1982).

The toxicological studies performed so far would suggest that the tolerance of man to Ro 03-8799 may prove to be twice that of MISO.

Improved tolerance, higher concentration in tumour and greater radiosensitizing efficiency may prove Ro 03-8799 to be a considerable improvement as a radiosensitizer when compared with misonidazole. Only a full-scale study will determine, however, whether this promise will be achieved. The evidence is certainly encouraging and a more extensive administration of the drug to man is being undertaken.

We wish to thank the Medical Research Council for the Programme Grant supporting the clinical work with radiosensitizers at Mount Vernon Hospital.

REFERENCES

Dische, S., Saunders, M. I. & Stratford, M. R. L. (1981*a*) Neurotoxicity with desmethylmisonidazole. *Br. J. Radiol.*, **54**, 156.

Dische, S., Saunders, M. I., Riley, P. J. & 4 others (1981*b*) The concentration of desmethylmisonidazole in human tumours and in cerebrospinal fluid. *Br. J. Cancer*, **43**, 344.

Rich, T. A., Dische, S., Saunders, M. I., Stratford, M. & Minchinton, A. (1981) A serial study of the concentration of misonidazole in human tumors correlated with histologic structure. *Int. J. Radiat. Oncol. Biol. Phys.*, **7**, 197.

Schwade, J. G., Strong, J. M. & Gangji, D. (1979) Intravenous misonidazole. *Int. J. Radiat. Oncol. Biol. Phys.*, **5**, 192.

Smithen, C. E., Clarke, E. D., Dale, J. A. & 4 others (1980) Novel (nitro-1-imidazolyl) alkanolamines as potential radiosensitizers with improved therapeutic properties. In *Radiation Sensitizers: Their Use in the Clinical Management of Cancer* (Ed. Brady). New York: Masson Publishing. p. 22.

Wardman, P. (1982) Molecular structure and biological activity of hypoxic cell radiosensitizers and hypoxic-specific cytotoxins. In *Advanced Topics of Hypoxic Cell Radiosensitizers* (Eds Adams *et al.*). New York: Plenum Press. p. 49.

Williams, M. V., Denekamp, J., Minchinton, A. and Stratford, M. R. L. (1982) *In vivo* assessment of basic 2-nitroimidazole radiosensitizers. *Br. J. Cancer*, **46**, 127.